# Population-specific facial traits and diagnosis accuracy of genetic and rare diseases in an admixed Colombian population

**DOI:** 10.1038/s41598-023-33374-x

**Published:** 2023-04-27

**Authors:** Luis M. Echeverry-Quiceno, Estephania Candelo, Eidith Gómez, Paula Solís, Diana Ramírez, Diana Ortiz, Alejandro González, Xavier Sevillano, Juan Carlos Cuéllar, Harry Pachajoa, Neus Martínez-Abadías

**Affiliations:** 1 Departament de Biologia Evolutiva, Ecologia i Ciències Ambientals (BEECA), Facultat de Biologia, Universitat de Barcelona (UB), Av. Diagonal, 643. Planta 2, 08028 Barcelona, Spain; 2 Centro de Investigaciones en Anomalías Congénitas y Enfermedades Raras (CIACER), Universidad ICESI, Cali, Colombia; 3 Servicio de Genética Clínica, Fundación Valle del Lili, Cali, Colombia; 4 HER - Human-Environment Research Group, La Salle - Universitat Ramon Llull, Barcelona, Spain; 5 Universidad ICESI, Cali, Colombia

**Keywords:** Skeleton, Diagnostic markers, Paediatric research

## Abstract

Up to 40% of rare disorders (RD) present facial dysmorphologies, and visual assessment is commonly used for clinical diagnosis. Quantitative approaches are more objective, but mostly rely on European descent populations, disregarding diverse population ancestry. Here, we assessed the facial phenotypes of Down (DS), Morquio (MS), Noonan (NS) and Neurofibromatosis type 1 (NF1) syndromes in a Latino-American population, recording the coordinates of 18 landmarks in 2D images from 79 controls and 51 patients. We quantified facial differences using Euclidean Distance Matrix Analysis, and assessed the diagnostic accuracy of Face2Gene, an automatic deep-learning algorithm. Individuals diagnosed with DS and MS presented severe phenotypes, with 58.2% and 65.4% of significantly different facial traits. The phenotype was milder in NS (47.7%) and non-significant in NF1 (11.4%). Each syndrome presented a characteristic dysmorphology pattern, supporting the diagnostic potential of facial biomarkers. However, population-specific traits were detected in the Colombian population. Diagnostic accuracy was 100% in DS, moderate in NS (66.7%) but lower in comparison to a European population (100%), and below 10% in MS and NF1. Moreover, admixed individuals showed lower facial gestalt similarities. Our results underscore that incorporating populations with Amerindian, African and European ancestry is crucial to improve diagnostic methods of rare disorders.

## Introduction

According to the Online Mendelian Inheritance in Man (OMIM) databank, there are more than 10,000 genetic and rare diseases (RD) affecting 7% of the world's population^1,2^. This corresponds to approximately 500 million people. Although as a whole genetic and RD are a significant cause of morbidity and mortality in the pediatric population^3^, by separate each disorder affects a very reduced number of people. Depending on the country, the prevalence to consider a disease as rare ranges from 1 affected individual in 50,000 people to 1 in 200,000. This low prevalence has limited the research on rare disorders.

Currently, there is limited knowledge on the etiology of these disorders. A reduced percentage of diseases (20%) presents a known molecular basis associated to a detailed phenotype description, and treatment is only available for 0.04% of RD^3^. As orphan diseases, many RD are chronic and incurable, representing severe and debilitating conditions^4^. The diagnosis and management of genetic RD is currently a clinical challenge^5^. Precise and early diagnosis is crucial for individuals and their families to get effective care and to reduce disease progression. However, due to the limited knowledge and complexity of these pathologies, diagnosis may take several years^6^. People often suffer during a long diagnostic odyssey, with delays in their correct treatment and management^7^. For most rare diseases, there are no reliable biomarkers for early diagnosis^8^.

Among the wide constellation of clinical symptoms associated to genetic and rare disorders, craniofacial dysmorphologies emerge as potential biomarkers^9,10^. These phenotypes are highly prevalent^2,6^ and are commonly used for diagnosis, management and treatment monitoring of genetic and RD^6^. Up to 40% of these disorders present characteristic craniofacial phenotypes, including Down, Morquio, Noonan, Apert, Rett, Fragile X, Williams-Beuren and Treacher-Collins and velocardiofacial syndromes, as well as other conditions such as microcephaly, holoprosencephaly, palate/lip cleft, and other 2,000 rare genetic disorders^10,11^.

The genetic and environmental factors causing these disorders alter the complex process that orchestrates facial morphogenesis during pre- and postnatal development, inducing facial dysmorphologies. Facial development is highly regulated by multiple signaling pathways^12–14^, including Fibroblast Growth Factor (*FGF*), Hedgehog (*HH*), Wingless (*WNT*) and Transforming Growth Factor Beta (*TGF-β*) and Bone Morphogenetic Proteins (*BPMs*). Disruptions in the regulation of any of these signaling pathways can lead to facial dysmorphogenesis^15^.

The facial patterns associated with each disorder are unique, but vary within and among diagnostics, ranging from subtle facial anomalies to severe malformations^16^. In the clinical practice, craniofacial dysmorphology is commonly assessed through qualitative visual assessment and basic anthropometric measurements. However, this approach may not capture with optimal precision the anatomical complexity of the facial dysmorphologies associated with these disorders. Qualitative descriptions of facial phenotypes are sometimes based on general terms such as coarse face, large and bulging head; saddle-like, flat bridged nose with broad, fleshy tip; or malformed teeth^17–19^. Accurate identification of dysmorphic features for diagnosis thus depends on the clinician’s expertise, and only highly trained dysmorphologists are able to recognize the facial “gestalt” characteristic of the rarest disorders^19^.

Recent research seeks to incorporate into the clinical diagnosis of RD the use of objective and quantitative tools to assess facial phenotypes^20–25^. Automated systems have been developed to improve and accelerate the diagnostic process^9,10,26^. Within the clinical practice, Face2Gene is the most commonly used system (FDNA Inc., https://www.face2gene.com/), a community-driven phenotyping platform trained over 17,000 people representing more than 200 syndromes^9^. Face recognition is performed on 2D images that can be collected with any type of digital camera or phone, without previous training. Syndrome classification is achieved using DeepGestalt, a cascade Deep Convolutional Neural Network (DCNN)-based method that achieved an average 91% top-10 accuracy in identifying the correct syndrome^9^.

Other diagnostic approaches based on 3D photogrammetry have been developed more recently^10,20,21^. The advantage of 3D facial models is that they are more efficient than 2D images in capturing the complexity of facial phenotypes, but their widespread use is limited because the photographic equipment required for generating 3D models is not commonly available in the clinical practice. Hallgrímsson et al. (2020)^10^ analyzed 3D facial models from 7,057 subjects including subjects with 396 different syndromes, relatives and unrelated unaffected subjects (https://www.facebase.org/). Deep phenotyping based on quantitative 3D facial imaging and machine learning presented a balanced accuracy of 73% for syndrome diagnosis^20^.

Automated methods have thus demonstrated high potential to facilitate the diagnosis of facial dysmorphic syndromes^6,9,10,26^. These tools present high accuracy diagnosis in European and North American populations, that are the populations in which the machine learning algorithms have been trained and validated. However, these tools have not been thoroughly tested in populations with different ancestries, and it is not well understood the how facial phenotypes associated with genetic and RD might be influenced by the complex patterns of population ancestry characterizing human populations.

### Population ancestry in facial dysmorphologies: a long-disregarded factor

Facial shape shows wide variation across world-wide human populations^27^. Facial differences between populations are detected in the shape of the forehead, brow ridges, eyes, nose, cheeks, mouth and jaw^28^. These facial phenotypes result from divergent evolutionary and adaptive histories of human populations occurred during the evolution of *Homo sapiens* over the last 200,000 years. Nowadays, continuous migration and admixture keep shaping the facial phenotypes of human populations. Depending on dominance and epistatic interactions between alleles fixed or predominant in each parental group^30^, admixed populations can display a variety of craniofacial morphologies, ranging from resemblance to one of the parental groups to a combination of both parental phenotypes and the evolution of novel phenotypes^29^. Therefore, the evolutionary and population dynamics of human populations result in genetic and phenotypic patterns that surrogate population ancestry^30–32^, and can modulate the facial phenotypes associated to disease.

Few studies to date have analyzed the craniofacial phenotypes associated with genetic and RD in populations of non-European descent^33–36^, leaving African, Asian and Latin-American populations often disregarded and underrepresented. Unfortunately, there are no reliable representations of facial phenotypes in genetic and rare diseases in populations of non-European descent. However, it is crucial to account for the influence of population ancestry on facial variation to develop quantitative approaches that efficiently diagnose these disorders in populations from all over the world.

To cover this gap, here we assessed the facial dysmorphologies associated to prevalent genetic and RD in a Latin-American population from the Southwest of Colombia. Latin-Americans are fascinating cases of hybrid/admixed populations that evolved over relatively short periods of time^30,37^. Peopling of the Americas likely started 12–18,000 years ago^38,39^ by migration waves coming from North and South East Asia^30^, following coastal and continental routes^41^. Amerindian populations established all over the continent and adapted to a variety of environments over thousands of years. During the last 600 years, admixture with European and African populations further shaped the genetic ancestry of Latin-American populations^42,43^. In particular, the population from the region of Cali is the result of diverse migratory processes^44^. Admixture with the indigenous Amerindian population began in the sixteenth century with the arrival of Spanish colonizers. In the eighteenth century, large colonial settlements of slaves brought from Africa were established in Cali for the exploitation of sugar cane that significantly changed the population structure of Valle del Cauca. Nowadays, the population of Cali is characterized by indigenous and mestizo communities, with Amerindian and African ancestry components predominating over the European ancestry contribution^44^.

In this study, we compared the facial phenotypes associated to four genetic and RD, including Down syndrome (DS), Mucopolysaccharidosis type IVA metabolic disorder known as Morquio syndrome (MS), and two types of RASopathies, Noonan syndrome (NS) and Neurofibromatosis type 1 (NF1). The facial phenotype of these syndromes has not been previously characterized in Latin-American populations, and differences between populations with different ancestry backgrounds have not been assessed^34–36^. Here, we quantitatively assessed the facial phenotypes associated to these syndromes, and compared our results in a Colombian admixed population with those reported in European descent populations. We also assessed the diagnostic accuracy of automatic methods currently used in the clinical practice, and detected evidence suggesting that further research is needed to optimize these methods in admixed populations of non-European descent.

## Materials and methods

### Participant recruitment for photographic sessions

The Colombian sample comprised 130 individuals from Valle del Cauca, a Southwest region in Colombia (Table 1). The cohort included 79 age matched controls and 51 individuals diagnosed with Down, Morquio, Noonan and Neurofibromatosis type 1 syndromes that were recruited from the clinical genetics consultation at Hospital-Fundación Valle del Lili in Cali (Colombia), a tertiary health reference center for these genetic and rare disorders. In most cases, clinical diagnoses were confirmed by molecular genetic testing.

**Table 1:** Sample composition by diagnosis. The table provides the number of male (M) and female (F) participants, as well as the total sample size for each syndrome. The age range within each diagnostic group is also provided, where ${\overline{\text{x}}}$ represents the average age.

Diagnosis	M	F	Total	Age (years old)
Control	32	47	79	4–59 (${\overline{\text{x}}}$ = 23.5)
Down syndrome	8	11	19	3–28 (${\overline{\text{x}}}$ = 12.7)
Morquio syndrome	6	5	11	9–28 (${\overline{\text{x}}}$ = 17.9)
Noonan syndrome	4	5	9	5–39 (${\overline{\text{x}}}$ = 16.4)
Neurofibromatosis type 1	6	6	12	6–52 (${\overline{\text{x}}}$ = 17.5)
**Total**	56	74	130	

Down syndrome (DS, OMIM 190685), caused by trisomy of chromosome 21, was selected because it is one of the most common genetic disorders, and previous studies have shown that the clinical manifestations associated with DS vary across ethnicities^35^. Within RD, we included Morquio syndrome type A (MS, OMIM 253000) because Colombia presents one of the highest prevalence of MS in the world, probably as a result of founder effects^45^. Morquio syndrome is a subtype of Mucopolysaccharidosis disorders caused by more than 180 autosomal recessive mutations in the *GALNS* gene^46^ that alter the metabolism of the extracellular matrix glycosaminoglycans^47^. Individuals with MS show coarse facies with an excessively rapid growth of the head^48^.

Finally, we also included in the analyses two RASopathies, Noonan syndrome (NS, OMIM 163950) and Neurofibromatosis type 1 (NF1, OMIM 162200), which are prevalent in Valle del Cauca and present altered craniofacial development by genetic mutations that cause Ras/MAPK pathway dysregulation^49^.

To assess the facial phenotypes associated with these disorders, individuals diagnosed with DS, MS, NS and NF1 and age matched controls were recruited for photographic sessions at educational and research centers in Cali (Colombia) in 2021. The photographic material was taken under the protocol approved by ethics committee “Human Research Ethics Committee of the Icesi University” with Approval Act No. 309. To photograph the participants and to record relevant clinical information, we obtained informed consent from the participants or from their parents or legal guardians in the case of minor children, in accordance with national guidelines and regulations.

### Facial image acquisition and anatomical landmark collection

Facial shape was captured from 2D images taken using a professional digital camera (SONY Alpha 58 + 18–55) that was attached to a tripod and placed at one-meter distance in front of the participants. To capture a natural facial gesture, the images were acquired in an upright position with facial neutral expression. Participants were asked to sit still, looking towards the front, with open eyes and closed mouth. Although this was challenging in children with Down syndrome, who usually show hyperactivity and tongue protrusion due to hypotonia, several photographs were taken until a neutral facial expression was achieved.

**Figure 1:**
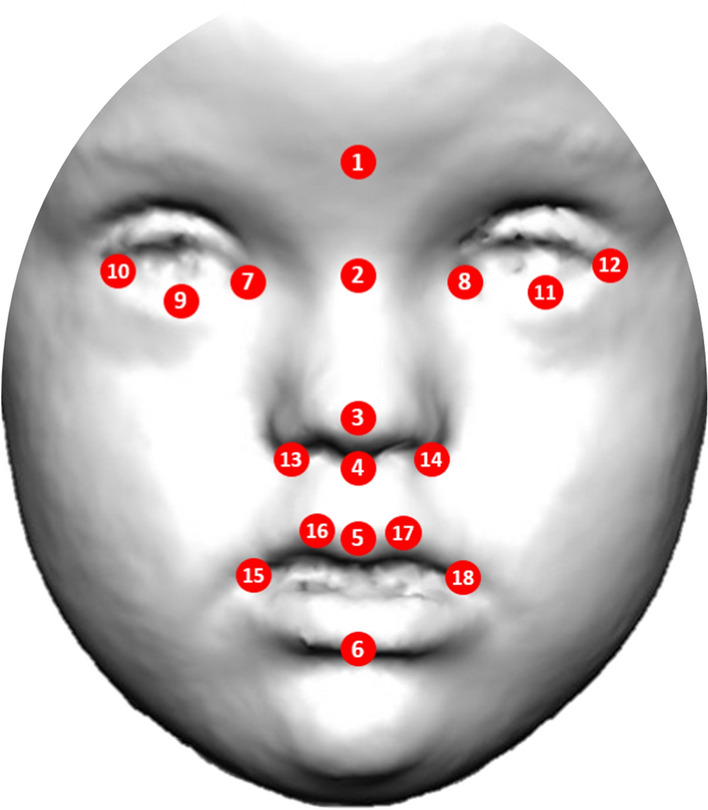
Anatomical position of facial landmarks used in morphometric and statistical analyses to quantify dysmorphologies associated to genetic and rare disorders Down, Morquio, Noonan syndromes and Neurofibromatosis type 1 in a Colombian population.

To measure facial shape of each individual and to detect the traits associated with each disorder, we recorded the 2D coordinates of a set of 18 anatomical facial landmarks (Fig. 1 and Supplementary Table 1). Landmarks were acquired using an automatic facial landmark detection procedure adapted from the open-source software library Dlib^50^. The automatic landmarking process is explained in detail in Supplementary Information. In brief, from the set of 68 landmarks registered by Dlib, 15 landmarks directly matched our configuration of 18 facial landmarks (Fig. 1, Fig. S1, Table S1). Three additional landmarks were approximated through direct computations between the landmarks coordinates automatically returned by Dlib: the glabella was computed as the midpoint point between the innermost points located in the eyebrows, and the palpebrale inferius landmarks of the right and left eyes were computed as the midpoint between the two central lower eyelid landmarks.

The validity of the data was assessed by comparing the coordinates of landmarks automatically detected by Dlib with the coordinates of landmarks manually collected by an expert facial morphologist. Manual and automatic measurement differences were assessed for each individual landmark using the root mean square error (RMSE) (Fig. S2). This method was first validated with the 2D facial images of 20 control subjects, and the average RMSE was 1.75 mm. To validate the automatic landmarking method with images of syndromic patients, we manually landmarked 20 patients, including 5 individuals diagnosed with each syndrome represented in our sample. The RMSE for syndromic patients was slightly higher (RMSE = 1.96 mm), but below 2 mm (Fig. S2). Considering that this error threshold is widely accepted in studies of biological anthropology for craniometric measurements^51^, the precision of the automatic detection method of anatomical points was validated on both control and syndromic samples.

### Quantification of facial phenotypes

We used Euclidean distance matrix analysis (EDMA) to describe the facial phenotype associated to each syndrome. EDMA is a robust morphometric method for assessing local differences between samples^52^ by detecting linear distances that significantly differ between pairwise sample contrasts and comparing patterns of significant differences across samples.

To account for size differences between subjects, the 2D coordinates of the facial landmarks of each subject were scaled by their centroid size, estimated as the square root of the sum of squared distances of all the landmarks from their centroid^53^. After scaling, as EDMA represents shape as a matrix of linear distances between all possible pairs of landmarks, a total of 153 unique facial measurements were calculated for each individual. Linear distances were compared for each group of DS, MS, NS and NF1 syndromes with control individuals by performing a two-tailed two-sample shape contrasts on all unique inter-landmark linear distances from each sample. Relative differences between patients and controls were computed as (mean distance in controls—mean distance in patients) / mean distance in controls.

Statistical significance was assessed using a non-parametric bootstrap test with 10,000 resamples. EDMA statistically evaluated the number of significant local linear distances in each two-sample comparison based on confidence interval testing. We used the default α level in EDMA (α = 0.10), and a 90% confidence interval was calculated for each linear distance. The shape differences were sorted in increasing order, and the first 5% and the last 5% differences were discarded. The resulting minimum and maximum differences were used to set up the lower and upper confidence limits for each linear distance. Interlandmark distances were considered non-significantly different between controls and patients when the resulting interval contained the value zero. Otherwise, the equality null hypothesis was not accepted, and we assumed that a significant shape difference existed at the α level^54^. To pinpoint specific local shape differences and to reveal the unique morphological pattern of variation associated with each disorder, the ten longest and shortest significant relative differences were plotted on facial figures.

### Facial dysmorphology score

To confirm that results were not random due to the small sample sizes available in rare diseases, we combined the results from EDMA with an iterative bootstrapping method that further assessed whether the facial dysmorphologies associated to each syndrome were statistically significant^55^. First, we estimated from the EDMA results a facial dysmorphology score (FDS) as the percentage of significantly different distances between patient and control groups. Then, we ran simulations with random samples of controls and patients generated by iterative bootstrapping to assess the statistical significance of the patterns revealed by EDMA. For each disorder, we first created subsamples of N randomly chosen controls (where N is the total number of patients available in the sample). Then, using a subsampling approach, we automatically generated random pseudo-subsamples containing a known number of patients (namely M). This procedure was repeated with increasing numbers of patients and resulted in a series of staggered pseudo sub-samples that contained from M = 0 to M = N patients. A total of 150 simulations were run in each round, and in each of these simulations, we computed an EDMA analysis and an FDS score.

The results from each round of random groups were separately represented in histograms. The first round of simulations contained no patients (M = 0) and only included control individuals, representing facial differences that can be found randomly in the general population. To assess whether the FDS value obtained using the complete patient dataset was significantly different or similar to the FDS resulting from a random sample, we compared the distribution of FDS random values with the FDS observed in the whole sample. The *P*-value assessing the statistical significance of the comparison was computed as the ratio between the number of simulations containing no patients that provided a higher FDS than the observed FDS divided by the total number of simulations. *P*-values below 0.05 indicated that the FDS obtained using the real dataset was higher that the FDS obtained randomly in a sample of control subjects.

### Face2Gene diagnostic assessment

To assess the accuracy of automated diagnostic methods in the Colombian sample, we compared the clinical diagnosis based on clinical and genetic testing with the diagnosis estimated from the frontal facial 2D images of the patients using the Face2Gene technology (FDNA Inc., Boston, MA, USA; https://www.face2gene.com). Following Gurovich^9^, we assessed the top-one and top-five accuracies for each disorder, estimated as the percentage of cases where the Face2Gene model predicted the correct syndrome as the first result or within the five first results from the sorted list of probable diagnoses. We also calculated these accuracies expanding the diagnostic range to the disorder family.

Moreover, we evaluated the similarity between the Colombian patients and the facial gestalt models used by Face2Gene for syndrome classification. For each individual, we selected the first diagnostic prediction that matched their clinical and genetic diagnosis and recorded the gestalt similarity. We classified the level of similarity between the individual and the corresponding gestalt model into seven categories, including “very low”, “low”, “low-medium”, “medium”, “medium–high”, “high”, and “very high” gestalt similarity, using the “gestalt level” barplot provided by Face2Gene.

Finally, to further test the influence of population ancestry on the diagnostic accuracy of Face2Gene, and to directly compare the results with individuals from European descent populations, we performed an extensive search of public image databases to obtain 2D photos of European subjects diagnosed with DS, NS, MS and NF1 syndromes. We collected the images of 45 subjects with DS^56^; and 24 diagnosed with NS^57^. Unfortunately, no 2D images of European individuals diagnosed with MS and NF1 were found publicly available. Using these images, we tested the accuracy of Face2Gene in DS and NS employing the same method previously described for the Colombian population. However, we could not use these publicly available images to perform EDMA and FDS analyses on the European samples, because the pictures were not taken under controlled conditions^56^, and diverse facial expression and head position would lead to bias in results of quantitative shape comparisons.

## Results

EDMA analyses showed that each syndrome presented a characteristic facial phenotype.

**Figure 2:**
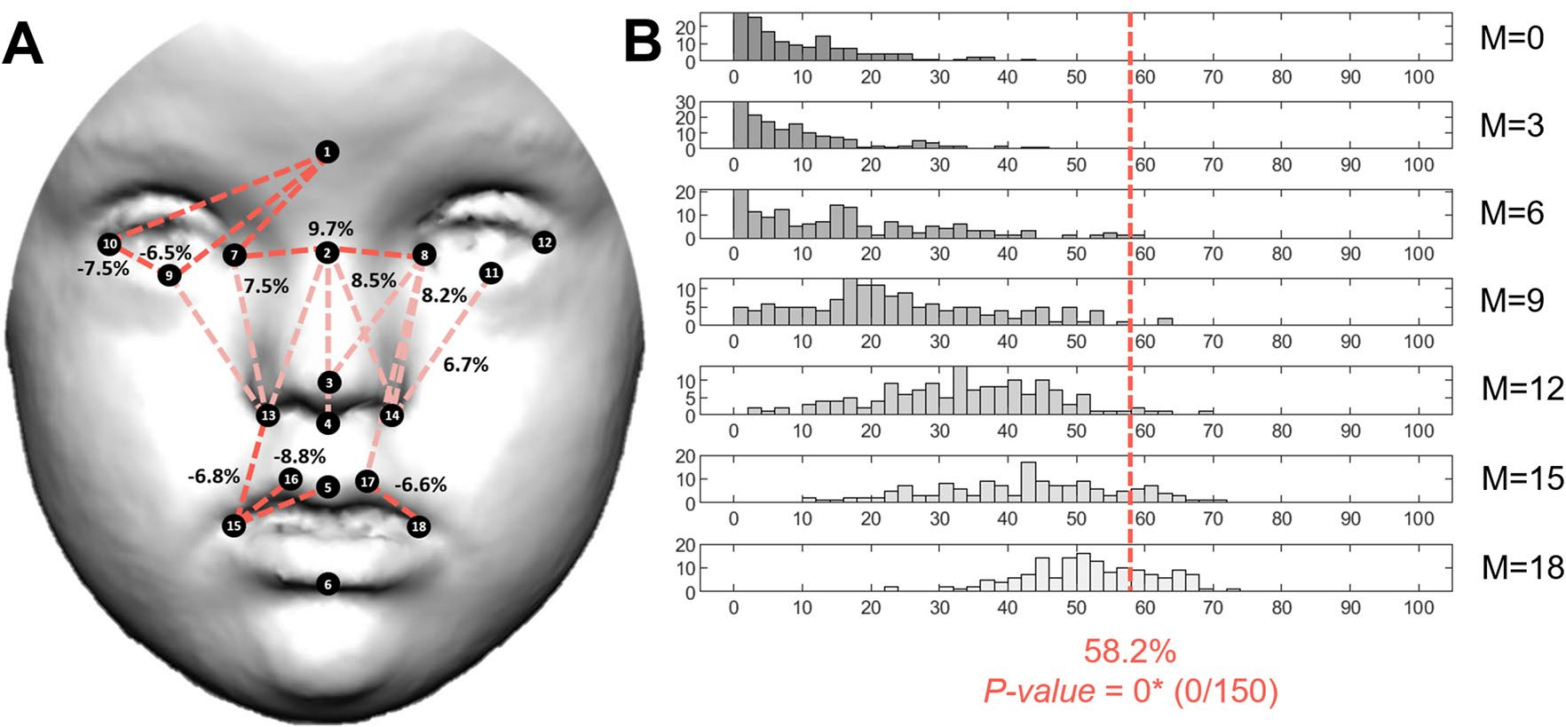
Localized Euclidean Distance Matrix Analysis facial shape pairwise contrasts and iterative bootstrapping tests of facial dysmorphology between controls and individuals diagnosed with Down syndrome. (**A**) EDMA results. Dotted lines represent facial measurements significantly different in control and patient groups. Lines in light tones indicate measurements that are shorter in patients as compared to controls, whereas lines in dark tones represent measurements that are longer in patients. (**B**) Iterative bootstrapping tests based on facial dysmorphology scores (FDS). Histograms represent the simulation results for each random group separately, which contain an increasing number of patients, from no patients (M = 0) to all patients (M = N). From top to bottom histograms, the simulations included 0, 3, 6, 9, 12, 15 and 18 patients. The dotted red line shows the FDS score obtained with the complete sample of control and patients (Table 1). * Statistically significant *P*-value.

In individuals with Down syndrome, all facial structures including the eyes, nose and mouth presented significant differences as compared to controls. Overall, DS was associated with wider but shorter facial traits (Fig. 2A).

Results showed a 6.5% increase of relative distance between the midpoint between the eyebrows (glabella) and the most inferior medial point of the lower right eyelid (palpelabre inferius), and a 7.5% increase between the right palpelabre inferius and the outer commissure of the right eyes (exocanthion), indicating hypertelorism. Additionally, in this Colombian sample, people with DS exhibited longer measurements in the buccal portion, with a 6–8% increase of mouth width as measured from the crista philtri to the chelions (Fig. 2A). However, the midfacial and nasal regions were reduced (Fig. 2A). People with DS presented a 6–8% reduction in measurements of midfacial height, with the largest difference detected as a 9.7% reduction of the distance between the tip and the root of the nose (Fig. 2A). The facial dysmorphology score (FDS) indicated that up to 58.2% of facial traits were significantly different in people with DS (Fig. 2B).

**Figure 3:**
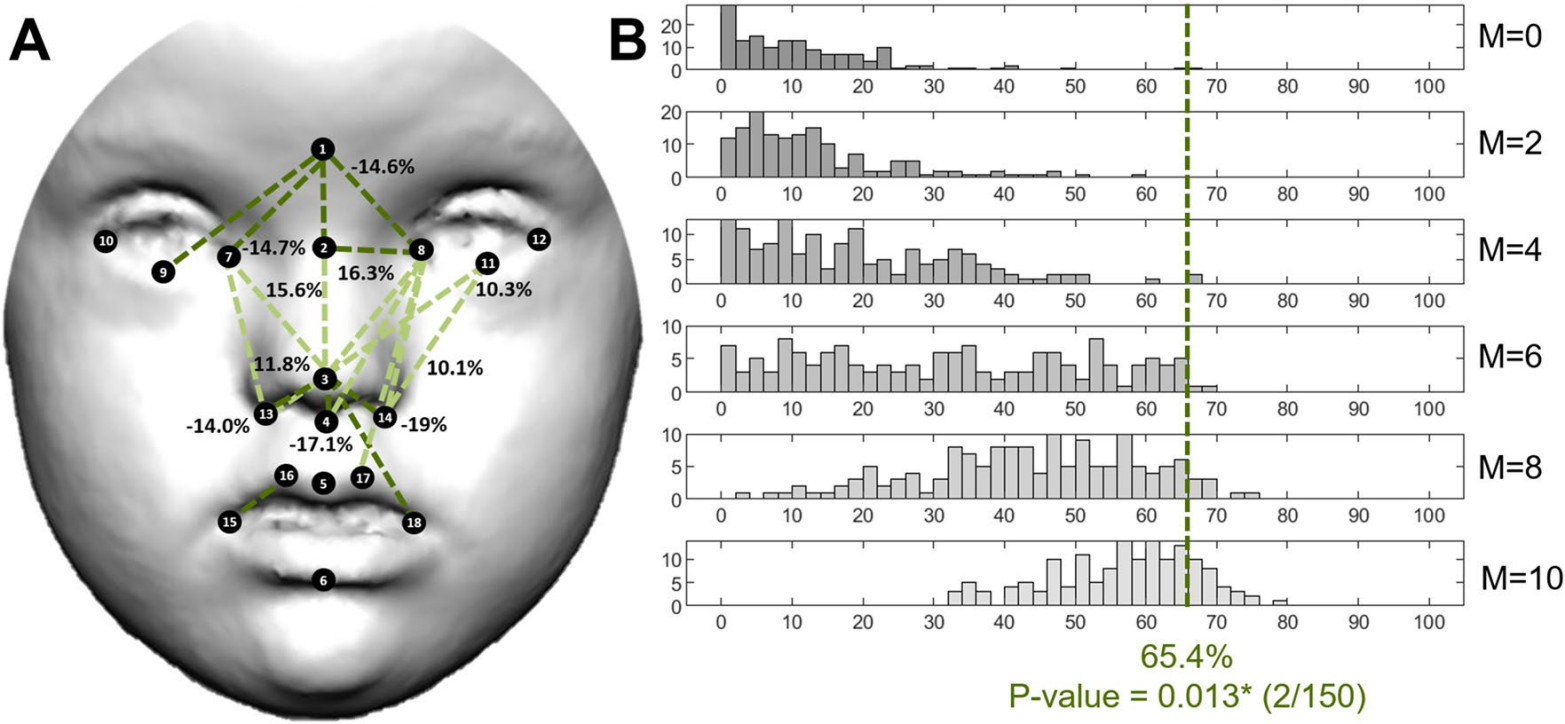
Localized Euclidean Distance Matrix Analysis facial shape pairwise contrasts and iterative bootstrapping tests of facial dysmorphology between controls and individuals diagnosed with Morquio syndrome. From top to bottom histograms, the simulations included 0, 2, 4, 6, 8 and 10 patients. For more details see legend in Fig. 2.

The facial pattern associated with Morquio syndrome was also characterized by wider and shorter midfacial traits, as observed in Down syndrome. However, facial dysmorphologies were more abundant and severe in MS than in DS, with 65.4% of facial traits significantly different in diagnosed individuals and higher percentages of relative change (Fig. 3 A, B). The most affected regions were the midface and the nose, whereas the mouth was the least affected. Individuals with MS presented hypertelorism, with 14% increase in the distance between the midpoint between the eyebrows (glabella) and the inner commissures of the left and right eyes (endocanthions). Individuals with MS also showed larger distances in the base of the nose, with a 14–19% increase in the distance from the tip of the nose to the insertion of the right and left alar bases (subalare) as compared to controls. Mouth width was also increased in MS; whereas midfacial heights measuring the distance between the eyes and the nose were significantly reduced from 10 to 16% in individuals with MS (Fig. 3A).

**Figure 4:**
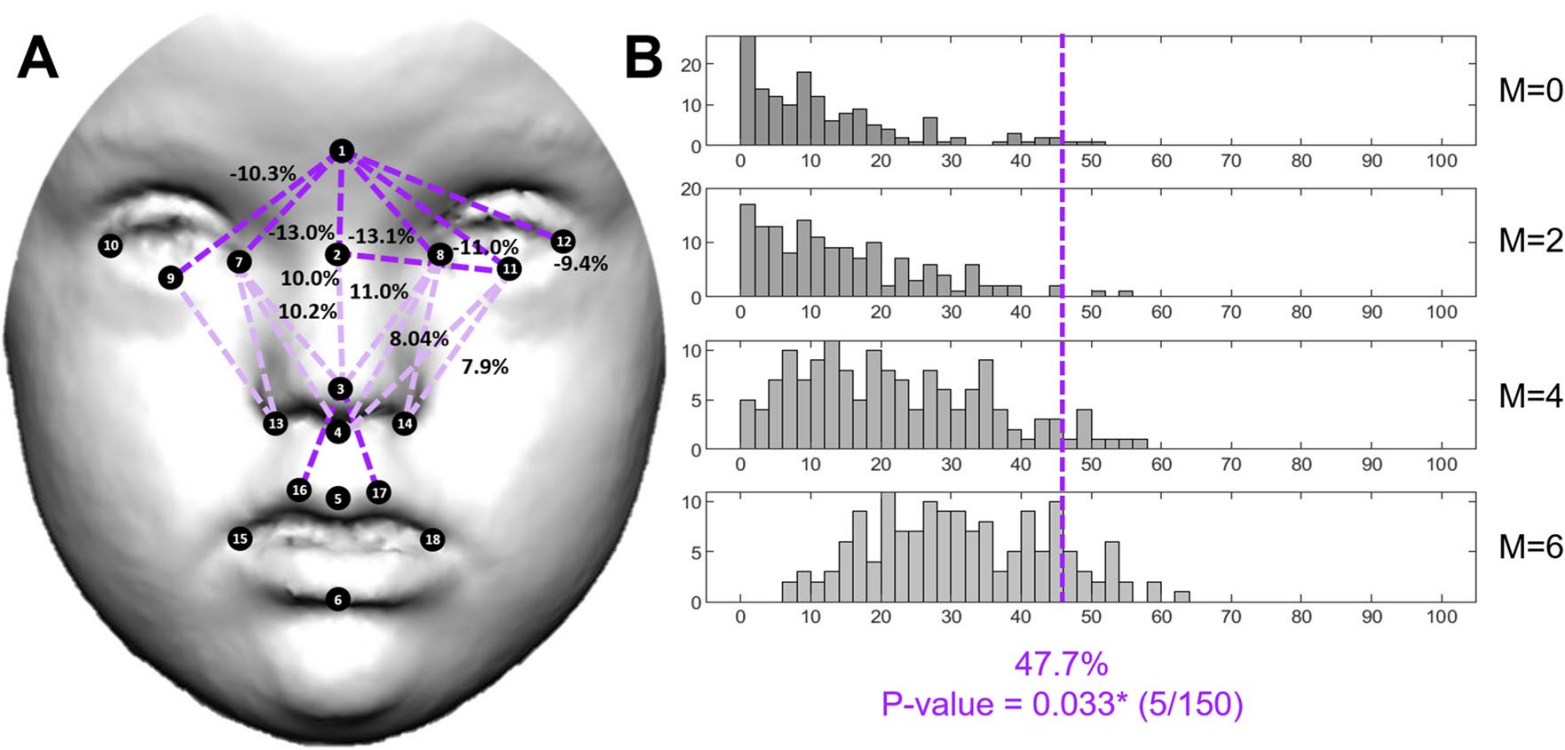
Localized Euclidean Distance Matrix Analysis facial shape pairwise contrasts and iterative bootstrapping tests of facial dysmorphology between controls and individuals diagnosed with Noonan syndrome. From top to bottom histograms, the simulations included 0, 2, 4 and 6 patients. For more details see legend in Fig. 2.

In Noonan syndrome, facial dysmorphologies were abundant and concentrated in the orbital and nasal regions. EDMA detected significantly increased distances in the upper face, but decreased distances in the midface (Fig. 4A).

Patients presented a lower position of the eyes, with 9 to 13% increased distances between the glabella or sellion and the landmarks located in the eyes. The mouth also showed a more inferior position, with 8–10% increased relative distance between the tip of the nose and the superior lip, but the shape of the mouth did not show large differences between patients and controls. The reduction of midfacial heights in individuals with NS ranged from 5 to 11%, with a similar magnitude as in DS (Fig. 4A). FDS indicated that 47.7% of facial traits were significantly different in NS (Fig. 4B).

**Figure 5:**
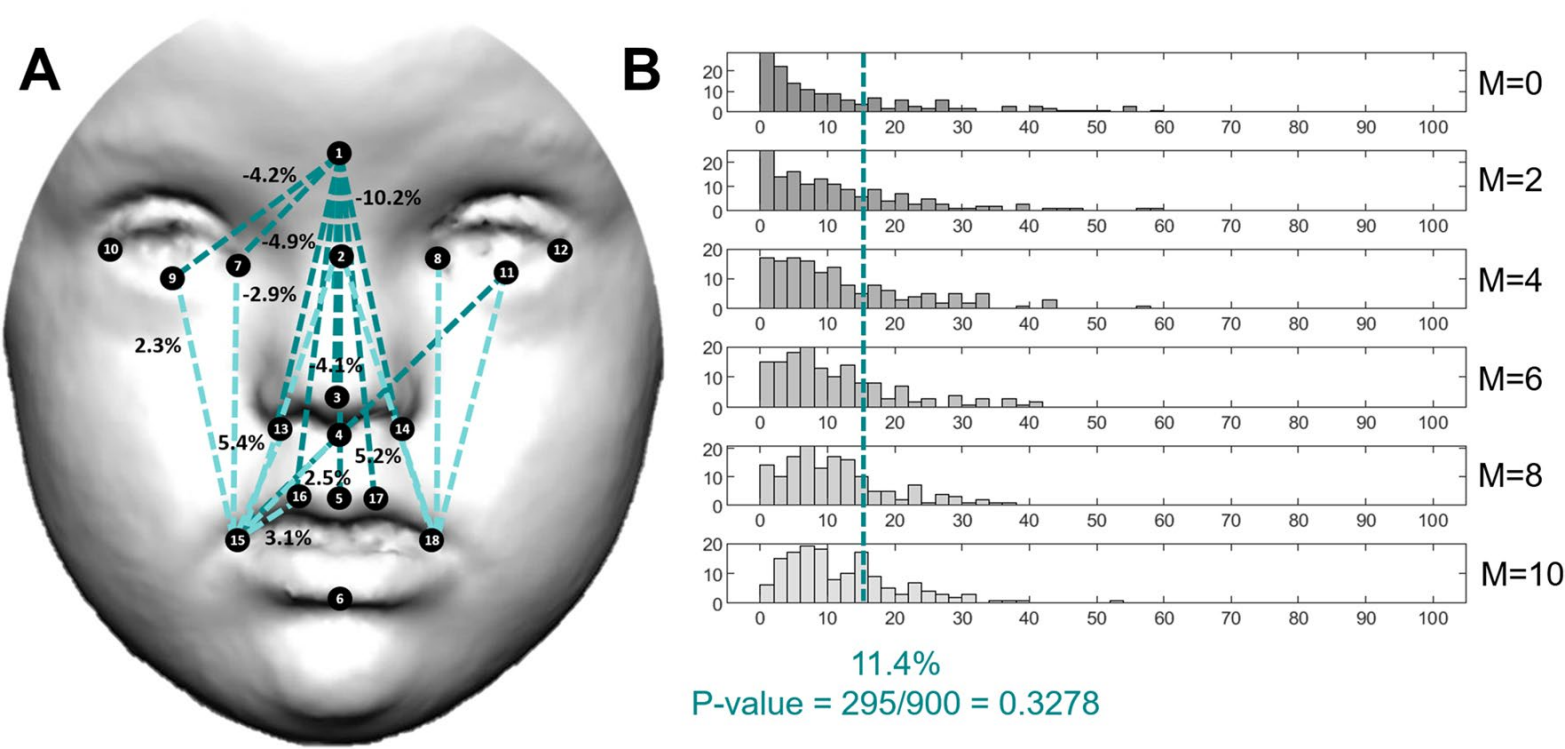
Localized Euclidean Distance Matrix Analysis facial shape pairwise contrasts and iterative bootstrapping tests of facial dysmorphology between controls and individuals diagnosed with Neurofibromatosis type 1. From top to bottom histograms, the simulations included 0, 2, 4, 6, 8 and 10 patients. For more details see legend Fig. 2.

Neurofibromatosis type 1 was associated with minor facial dysmorphologies, which were less abundant and less severe than in the previous syndromes (Fig. 5A). Individuals with NS only presented 11.4% of significantly different facial traits as compared to controls, and the percentages of relative change were low, mostly ranging from 1 to 5% (Fig. 5A,B). The largest difference was a 10% increase in facial distance between the glabella and the labiale superius (Fig. 5A). Along with larger distances in the midline of the face, EDMA detected reduced distances on the right and left sides of the face, with shorter distances from the right and left chelion to the eye landmarks, the endocanthion and the palpebrale inferius. Hypertelorism was not present in individuals with NF1 (Fig. 5A). In NF1, the FDS score was not significant (Fig. 5B), indicating that the facial dysmorphology pattern associated with NF1 is so subtle that overall is not larger than facial differences that could be randomly detected using a sample of control subjects.

For the other syndromes, the simulation tests confirmed that the facial dysmorphologies associated with Down, Morquio and Noonan syndromes were significant and different from random comparisons in control subjects. Few simulations resulted in a higher FDS than the FDS obtained with the complete real sample (Figs. 2B, 3B, 4B, first row and blue line). Moreover, in DS, MS and NS, facial dysmorphology scores increased as larger numbers of diagnosed individuals were included in the simulations (Figs. 2B, 3B, 4B, middle rows), confirming the severity of the facial dysmorphologies associated to these syndromes. Finally, the simulations comparing all recruited diagnosed individuals (last row) with random subsamples of control subjects (first row) indicated that FDS scores can range widely from 10 to 80%, underscoring the biasing effects of small sample sizes.

### Face2Gene accuracy in Colombian and European populations

After quantifying the facial dysmorphologies associated to DS, MS, NS and NF1 in the Colombian sample, we tested the accuracy of the diagnosis provided by the automatic diagnostic algorithms of Face2Gene. We assessed the correspondence between the estimated Face2Gene diagnosis based on facial frontal 2D images with the diagnosis based on clinical and genetic testing.

**Figure 6:**
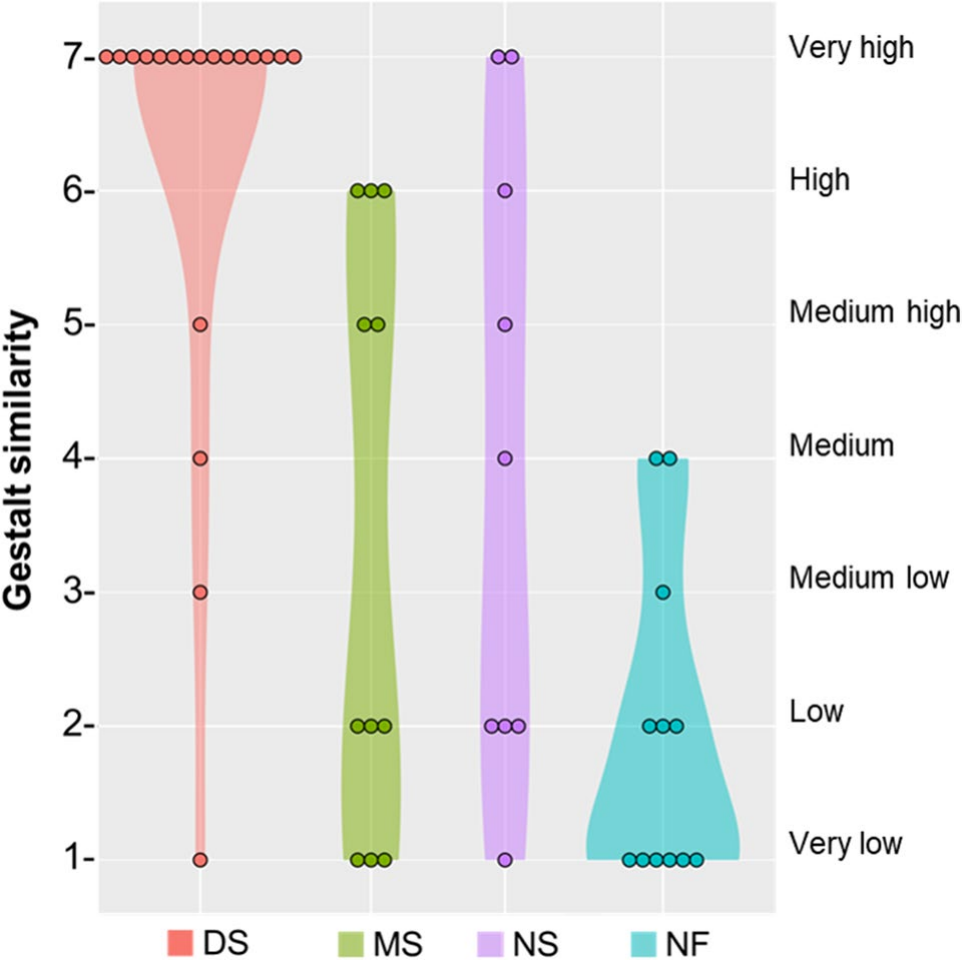
Gestalt similarity scores between Colombian individuals and Face2Gene models of Down, Morquio, Noonan and Neurofibromatosis type 1 syndromes. Violin plots are based on top-5 accuracy Face2Gene predictions within family disorder. Each plot shows the number of individuals scored at each gestalt similarity level, from very high to very low.

**Figure 7:**
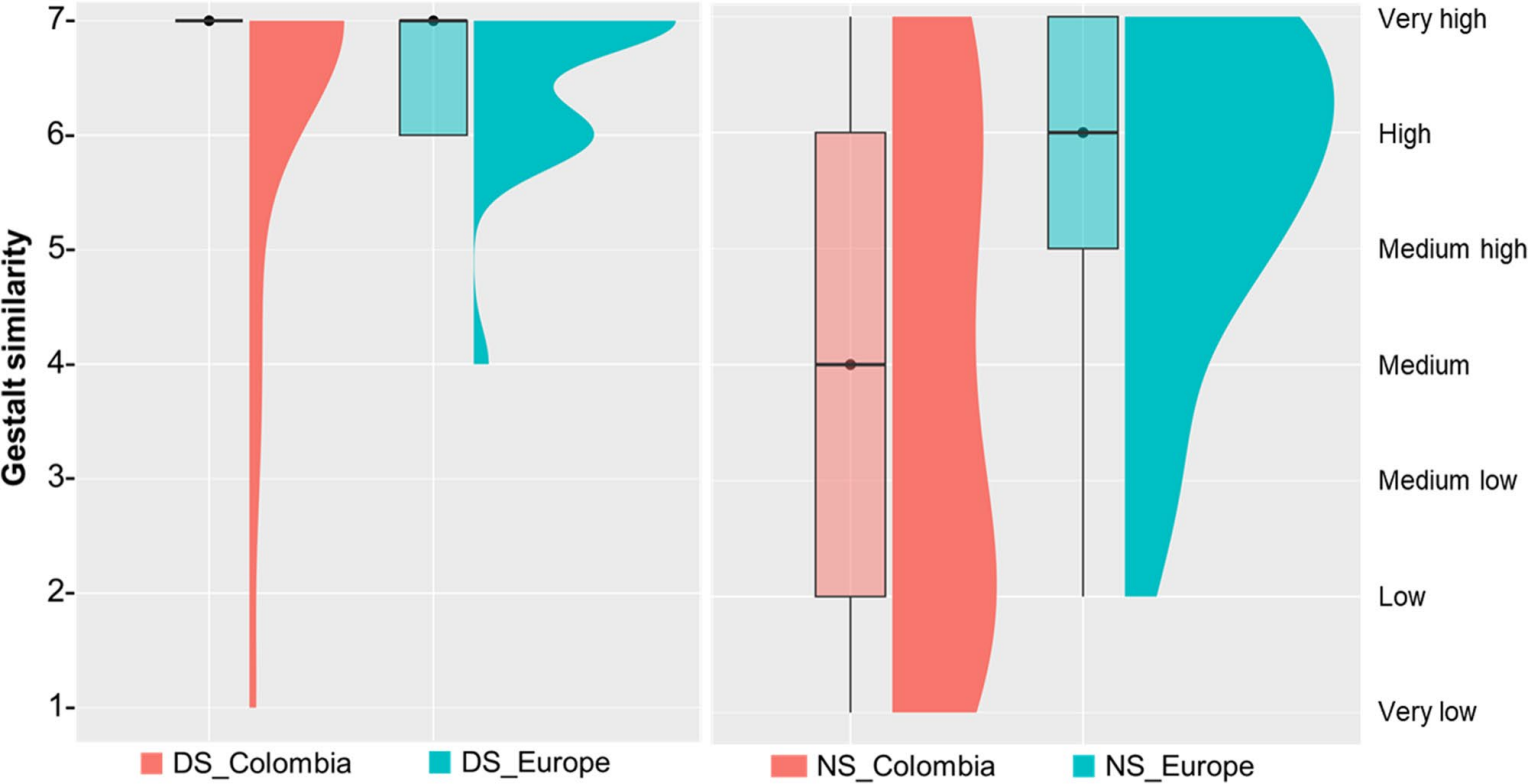
Comparison of gestalt similarity scores between Colombian and European populations in Down and Noonan syndromes. Raincloud plots are based on top-5 accuracy Face2Gene predictions within family disorder, and show the corresponding average gestalt similarity score, the range of variation, and the distribution within each disorder and population.

Face2Gene estimated Down syndrome diagnosis with top-1 accuracy of 100%, as DS diagnosis was listed as the first diagnosis in all individuals, with an average gestalt similarity of 6.2 (Table 2, Fig. 6). When comparing the gestalt similarities in Colombian and European populations, a Wilcoxon test did not find a significant difference between the average gestalt similarity (*P* = 0.4). However, a Levene test detected a significant difference in the variance of gestalt similarity scores (*P* = 0.01). Whereas in the Colombian population the gestalt similarity in DS ranged from very high to very low; in the European population the range of variation was limited from very high to medium (Fig. 7).

**Table 2:** Accuracy of Face2Gene diagnosis based on 2D facial images in Down, Morquio, Noonan and Neurofibromatosis type 1 syndromes in a Colombian population. Percentage of cases matching the genetic diagnosis are provided for each syndrome, as well as gestalt similarity values.

	Top-1 accuracy				Top-5 accuracy			
	Exact diagnosis		Within disorder family		Exact diagnosis		Within disorder family	
	% cases	Gestalt similarity	% cases	Gestalt similarity	% cases	Gestalt similarity	% cases	Gestalt similarity
DS	100	6.2	100	6.2	100	6.2	100	6.2
MS	0	0	36.4	5.6	45.4	3	100	3.4
NS	66.7	5.2	66.7	5.2	77.8	4.7	88.9	4.4
NF1	8.3	1	50	1.7	66.6	1.2	66.6	1.6

In Morquio syndrome, the top-1 accuracy of Face2Gene was 0%, as the specific diagnostic of mucopolysaccharidosis type IVA (MPSIVA) was never listed as a first prediction (Table 2). Although Face2Gene could not identify the specific type of MS, the automatic diagnostic algorithms associated the facial dysmorphologies with a diagnosis related with mucopolysaccharidosis disorders in 36.4% of cases, with a medium–high average gestalt similarity of 5.6 (Table 2). When the first 5 diagnostic predictions were considered, the top-5 accuracy raised to 45.4% for exact MPSIVA diagnosis and to 100% for mucopolysaccharidosis disorders, but with a low-medium gestalt similarity (Table 2, Fig. 6). In our sample, we detected four genetic variants (p.Gly301Cys, p.Arg386Cys, p.Arg94Cys, p.Gly333Asp, and p.Ser80Leu) that are missense mutations commonly found in the Colombian population^45^ (Table S2). Due to the small sample size and genetic heterogeneity of the patients, it was not possible to test whether different genetic variants were associated to different facial phenotypes. Comparative European samples were not available.

The top-1 accuracy of Face2Gene for Noonan syndrome was 66.7%, with a medium–high average gestalt similarity of 5.2 when considering subjects in which the diagnosis was successful (Table 2). Top-5 accuracy increased to 77.8% for exact NS diagnosis, and to 88.9% when considering Noonan Syndrome-Like Disorder diagnoses, with a medium gestalt similarity of 4.4 and wide variation among individuals (Table 2, Fig. 6). Although differences did not reach statistical significance probably due to small sample sizes (*P* = 0.09), the comparison between populations showed that in Europe, both the diagnostic accuracy and the gestalt similarity were higher than in Colombia. Using 2D images of patients from European origin, the Face2Gene top-1 accuracy for NS was 100% and the average gestalt similarity was 5.5 (Fig. 7).

Finally, in Neurofibromatosis type 1, Face2Gene presented a top-1 accuracy of 8.3% associated with a very low gestalt similarity of 1 (Table 2). When diagnoses within the RASopathies disorder family were considered, 5 out of 12 individuals were diagnosed as Noonan syndrome and the top-1 accuracy raised to 50% (Table 2). The top-5 diagnostic accuracy was 66.6% and was associated with low gestalt similarity values of between 1 and 2 in 87.5% of individuals (Table 2, Fig. 6). Comparative European samples were not available for NF1.

## Discussion

Our analyses provided an accurate quantitative comparison of facial dysmorphologies in Down, Morquio Noonan and Neurofibromatosis type 1 syndromes in a Latin-American population from Colombia. An objective and highly detailed description of the facial phenotype is a major improvement over qualitative descriptions of the complex facial dysmorphologies associated with these genetic disorders. We quantified local facial trait differences presented in people diagnosed with these disorders as compared with age matched controls of the same population, localizing the largest statistically significant facial dysmorphologies.

Our results indicated differential facial patterns associated with each disorder, with major significant dysmorphologies in DS, MS and NS, and minor facial dysmorphologies associated with NF1. Different types of genetic alterations, which ranged from aneuploidy and overall genetic imbalance in DS; to point genetic mutations affecting different processes or signaling pathways, such as the metabolism of mucopolysaccharides in MS, and the *RAS/MAPK* pathway in NS and NF1, significantly affected the facial phenotypes. These genetic alterations deviate the signaling pathways regulating normal facial development^16,58^, and alter normal morphogenesis and growth during pre- and postnatal development^15^ of individuals with genetic and rare disorders.

### Population-specific facial traits in Colombian individuals with genetic and rare disorders

Overall, the facial patterns observed in the Colombian Latin-American population coincide with the descriptions reported in the literature for each syndrome ^48,59–61^. However, there are specific local traits that differ, suggesting that facial traits associated to genetic and rare diseases might be modulated by population ancestry, as a result of different evolutionary and adaptive histories of human populations^33–35^.

#### Down syndrome

Down syndrome presents a worldwide prevalence of 14 per 10,000 live births, with life expectancy increasing from 25 to 60 years in developing countries^62–65^. In most Latino-American regions, the real incidence of patients with DS remains unknown, and is usually underreported. A cross-sectional study in Brazil reported a DS birth rate of 4 cases per 10,000 live births^66^; whereas in Colombia several studies have reported a prevalence rate between 1 per 1,000 to 5 per 10,000 live births^67,68^. DS is an aneuploidy caused by trisomy of chromosome 21, and is the leading genetic cause of intellectual disability^63^. Moreover, DS is associated with craniofacial dysmorphologies that impair vital functions such as breathing, eating, and speaking. In the literature, the DS craniofacial phenotype is mostly based on the analysis of European descent populations, and the characteristic traits include brachycephalic heads with maxillary hypoplasia leading to facial flatness; depressed nasal bridge and reduced airway passages^59^; dysplastic ears with lobe absence; eyes with oblique palpebral fissures, epicanthal folds, strabismus and nystagmus^16,69^; and oral alterations including open mouth, cleft lip, lingual furrows and protrusion, macroglossia, micrognathia, and narrow palate^70,71^.

In the Colombian population, we found facial dysmorphologies that are consistent with the craniofacial patterns reported in the literature. For instance, our analyses detected differences in linear facial measurements that correspond to typical DS traits such as hypertelorism, maxillary hypoplasia, and shorter and wider faces associated to a brachycephalic head^16,72^. Results also suggested other characteristic traits of DS, such as midfacial retrusion, and depressed nasal bridge^59^. Open mouth and macroglossia^70,71^ were also observed during the photographic sessions in the participants of our study.

However, in contrast to European and North American populations^55^, in the Colombian population we detected that the mouth was wider in individuals diagnosed with DS as compared to euploid controls. This difference could be caused by unnatural facial gestures of the participants when asked to close the mouth during the photo shoot, or by facial differences associated to ancestry. In fact, Kruszka et al.^33–35^ analyzed individuals diagnosed with DS in diverse populations, and showed craniofacial differences between individuals from different populations (Africans, Asians, and Latin Americans), demonstrating that ancestry is a relevant factor when assessing craniofacial variation associated to rare disorders.

#### Morquio syndrome

In Morquio syndrome, the worldwide prevalence ranges from 1 case per 75,000 to 1 per 200,000 live births; whereas in Colombia the prevalence rises up to 0.68 per 100,000 live births^45^. As a mucopolysaccharidosis syndrome, the typical alterations of MS involve the supporting tissue and the osteoarticular system^73^. Individuals with MS display abnormalities such as skeletal dysplasia, short stature and trunk, kyphoscoliosis, pectus carinatum, genu valgum, and joint hyperlaxity^74^. Oral diseases often include periodontal disease, malocclusions, caries, and premature tooth loss^46^. Individuals with MS show coarse facies, with an excessively rapid growth of the head^48^. Craniofacial features include a prominent forehead, hypertelorism, prognathism, wide mouth and nose, depressed nasal bridge, plump cheeks, and lips with an oversized tongue^48^. In the Colombian population, the facial dysmorphologies observed were consistent with traits reported in the literature, which included hypertelorism, prognathism, wide nose, and wide mouth^46,48^.

In the Colombian sample, Morquio syndrome was associated with the most severe facial dysmorphologies. Considering that keratan and chondroitin sulfate alterations associated with MS cause irreparable damage to leukocytes and fibroblasts, and accumulate over life inducing extreme deformations of the osteoarticular system, facial dysmorphologies associated with MS are expected to increase with age, becoming more severe in adult individuals^46^. Further research is required to test this hypothesis and to assess whether pharmacological treatments can slow down the progression of the disease and reduce the facial dysmorphologies associated with MS. This is especially relevant in Colombia, which is a country with one of the highest prevalence of MS in the world^45^.

Moreover, dysmorphologies associated with MS vary among individuals. Typically, MS patients present severe phenotypes, although less severe forms have been described as mild or attenuated phenotypes^73^. There is no consistent evidence regarding the genotype–phenotype correlation in MS, and whether different *GALNS* mutations are associated with the degree of severity in facial dysmorphology. In our Colombian sample, we detected four genetic variants (*p.Gly301Cys*, *p.Arg386Cys*, *p.Arg94Cys*, *p.Gly333Asp*, and *p.Ser80Leu*). Two of these genetic variants, *p.Gly301Cys* and *p.Arg386Cys*, that are the most frequently reported mutations in cases of Morquio syndrome; specifically in Colombia, but also in other American (Brazil, Chile, Argentina, Canada), and European countries (Spain, Portugal, Italy, Poland)^45,75–77^. The high prevalence of the *p.Gly301Cys* mutation in the Colombian population could result from founder and migration effects^45^. The *p.Arg386Cys* variant has been further detected in China and Turkey^75–77^; whereas the *p.Arg94Cys* allele has been previously reported in Middle East, Brazil, and Italy^76,77^. Other genetic variants, such as *p.Ile113Phe*, which are more frequently reported in British and Irish populations^45,75–77^, were not detected in our Colombian sample. Further tests with larger samples associated to each genotype are needed to test whether the population-specific genetic variants can be associated to different facial phenotypes in Morquio syndrome.

#### RASopaties: Noonan and NF1 syndromes

Regarding Noonan syndrome, the worldwide prevalence of NS is 1 per 1,000 to 1 per 2,500 live births^49^. NS is the most common type of RASopathy, and is a rare genetically heterogeneous autosomal dominant disorder caused by mutations in either the *PTPN11*, *SOS 1*, *KRAS*, *BRAF* or *RAF1* genes. Individuals with NS display facial features such as hypertelorism, epicanthic folds, strabismus, downward slanting palpebral fissures, ptosis, high arched palate, deeply grooved philtrum with high peaks of upper lip vermillion border, midfacial hypoplasia and micrognathia, broad flat nose, low-set posteriorly rotated ears, curly/sparse/coarse hair, and short webbed neck^60^. In the Colombian population, we detected hypertelorism, downward slanting palpebral fissures, and midfacial hypoplasia in cases of NS, as reported in populations of European descent^60^. In addition, our results quantified relative changes in the position of the mouth in Colombian individuals diagnosed with NS not reported before^78^.

In Neurofibromatosis type 1, the worldwide incidence is 1 per 2,500 to 1 per 3,000 individuals^49^. NF1 is an autosomal dominantly inherited neurocutaneous disorder caused by a mutation in the *neurofibromin* gene. The clinical manifestations of NF1 are variable, and the timing of the onset has a major influence^49^. Regarding craniofacial traits, individuals with NF1 present macrocephaly, facial asymmetry caused by dysplasia of the sphenoid wings^61^, as well as bone deformities caused by plexiform neurofibromas, enlarged mandibular canal, retrognathic mandible and maxilla, and short cranial base^79^. The facial pattern associated with NF1 in individuals from Colombia was also compatible with typical traits of NF1, such as midface hypoplasia^49^. However, our results did not detect facial asymmetry or hypertelorism as prominent facial differences between diagnosed individuals and controls in the Colombian population^49^.

Overall, our results support previous evidence demonstrating that rare disorders present distinctive facial traits that are population specific, with clinical features that are significantly different in Africans, Asians, and Latin Americans^34–36^. However, comparative facial quantitative analyses including subjects from different world regions are not usually available for most genetic and rare disorders, and reference data for diagnosis is mainly based on phenotypes defined on populations of European descent. In fact, almost no images of individuals of Latin American origin are included in reference medical texts^16^. Our results underscore the need to extend the analyses to populations from all over the world to achieve a complete and more accurate phenotypic representation of genetic and RD to optimize the diagnostic potential of facial biomarkers in the clinical practice.

### Variable accuracy diagnosis in a Colombian population with diverse ancestry

Deep learning algorithms such as Face2Gene have shown potential as a reliable and precise tool for genetic diagnosis by image recognition^9,26,80,81^. In the Colombian sample analyzed here, Face2Gene diagnosed Down syndrome with 100% accuracy, with the same accuracy as in the European sample. This result suggests that in a relatively common genetic disorder such as DS, in which the machine learning algorithm is likely trained in a large sample of individuals with a distinctive and well-represented facial phenotype, Face2Gene shows high diagnostic accuracy, independently from the genetic ancestry.

However, we found that this result cannot be extrapolated to other rare disorders. For instance, we detected a lower accuracy in the diagnosis of Noonan syndrome in the Colombian sample as compared with the European sample. Although Face2Gene correctly identified the disorder in most Colombian subjects, especially when considering the top5-accuracy within Noonan syndrome-like disorders (88.9%), the percentage of top1-accuracy was reduced from 100% to 66.7% in the Colombian sample. We hypothesize that when machine learning algorithms are trained in a relatively small sample of individuals with homogeneous European ancestry, the accuracy of diagnosing rare disorders might be more sensitive to population ancestry. Individuals from diverse populations may show lower gestalt similarity scores when assessed with predictive models that are trained on a population with different genetic and facial variation, and this may lead to reduced diagnostic accuracy.

Unfortunately, no data was publicly available on European samples to compare the diagnostic accuracy of Face2Gene in Morquio and Neurofibromatosis type 1 syndromes. Our results showed that the top1-accuracy for exact diagnosis of Mucopolysaccharidosis type IVA was 0% in the Colombian sample, despite Morquio syndrome was associated with the most severe facial dysmorphologies. Only a low percentage of cases (36.4%) were identified as a mucopolysaccharidosis-like syndrome in the first prediction. In the case of NF1, the top1-accuracy was also very low (8.3%), although the facial dysmorphologies in this disorder were less abundant and severe, and this result could just reflect the difficulty to diagnose NF1 from facial traits.

Finally, in the Colombian sample we detected a wide range of variation in gestalt similarity scores for most disorders, even for Down syndrome. In European subjects, the gestalt similarity for DS was high or very high in 95.5% of cases, and only 5% of subjects showed a medium gestalt score, even when the images included in Ferry et al. (2020)^56^ were ordinary photos with uncontrolled lighting, pose, and image quality. In Colombia, 79% of individuals diagnosed with DS were associated with very high gestalt similarity values, but in 21% of subjects the gestalt similarity was lower, and ranged from medium–high to very low values. Specifically, individuals with the lowest scores exhibited traits that suggested an admixed ancestry, a hypothesis that needs further assessment.

### The potential of facial biomarkers to diagnose genetic and rare disorders

Qualitative visual assessment of facial dysmorphologies is frequently employed for diagnosis, clinical management and treatment monitoring of RD^16^. Experts in dysmorphologies can identify the facial “gestalt” distinctive of many dysmorphic syndromes^16^. However, this facial assessment relies on the expertise of the clinician, and is very challenging because there is no clear one-to-one correspondence between disorders and facial dysmorphologies. Different genetic mutations can cause the same syndrome or similar phenotypes, whereas the same mutation can induce different phenotypes^12,82^. In addition, within the same rare disease there may be several subtypes, and symptoms may vary even within individuals of the same genetic disorder and the same family^3^. This complex biology generates confusion at the time of diagnosis and warrants the development of efficient, objective and reliable diagnostic methods.

Computer-assisted phenotyping can overcome these pitfalls and provide widely accessible technologies for quick syndrome screening^6^. In this automated approach, methods can be based on 2D or 3D images^9,10,26^. The advantage of 2D methods is that data collection is easy and can be readily translated into the clinical practice, as physicians can take facial images even with simple digital cameras or smartphones. The collection of 3D models is more sophisticated and requires specialized equipment but provides more accurate phenotype descriptions by incorporating the depth dimension.

To further improve the methods of craniofacial assessment to diagnose individuals with genetic syndromes and RD that exhibit facial dysmorphologies, it is crucial to assess the large morphological variation displayed by human populations in facial phenotypes. Factors such as age, sex and ancestry should be accounted for in diagnostic methods. Clinical manifestations in some genetic disorders usually begin at an early age, with two thirds of patients expressing symptoms before the second year of birth^3^; although in other disorders facial dysmorphologies develop later, during postnatal development. Male and female faces present sexual dimorphism at adulthood^83^, and diseases can differently affect the facial phenotype depending on sex differences^84^.

The role of population ancestry in the facial phenotype associated with genetic and rare disorders also needs to be further investigated in future analyses, assessing the reliability and validity of automatic diagnostic tools in admixed populations with diverse contributions of Amerindian, African and European ancestry components. This is critical in rare disorders with heterogenous clinical presentation and phenotype, where clinical diagnosis is a challenging process^5,6^ that may take several years, leading to the so-called diagnostic odyssey^7^.

Accurate and early diagnosis of genetic and rare disorders are crucial for adequate health care and clinical management. Without a diagnosis, individuals and their families must proceed without basic information regarding their health and future developmental outcomes^6^. Even though gene-based technologies have greatly improved diagnostic procedures^25^, the mutations causing many rare diseases are still not known and access to genetic testing is limited^3^. Genetic consultations may become a long process, and broad molecular testing such as exome and genome sequencing represent a high expense that is not affordable for all families and health care systems, especially in low-medium income countries^7^. In this context, faster, non-invasive and low-cost diagnostic methods based on facial phenotypes emerge as complementary tools for providing earlier first reliable diagnoses^9,10,25,26^.

Therefore, in future research the recruitment of participants must be expanded to include as many individuals with RD as possible, together with large comparative samples of age-matched controls, from both sexes, and from diverse world regions that faithfully represent the complex craniofacial variation and evolutionary histories of human populations. For instance, the population in Southwestern Colombia is characterized by high levels of admixture from people with Native American, African, and European ancestry^44,85^. Including the morphological variation of faces from such different ancestry backgrounds is key to pinpoint the facial dysmorphologies associated with diseases in worldwide diverse populations^86^. Our simulation analyses further highlight the importance of maximizing the recruitment of diagnosed and control individuals, as results considerably change depending on the cohort and sample sizes.

## Conclusions

Facial phenotypes associated with genetic and rare disorders can be influenced by population ancestry^34–36^. Our ancestry comparisons highlight that diverse genetic background variation can modulate the phenotypic response to disease, affecting the accuracy of current tools of clinical diagnosis. In the future, deep learning algorithms including a high variety of populations with different ancestry backgrounds will optimize the precision and accuracy of diagnosis in an unbiased approach. Such predictive models will support clinicians in decision-making across the world.

## Supplementary Information

Supplementary Information.

## Data Availability

Raw phenotype data from the Colombian population cannot be made available due to restrictions imposed by the ethics approval. Images from publicly available sources can be accessed from the original publications^56,57^. Anonymized landmark data and Matlab code for computing Facial Dysmorphology Score (FDS) is available at https://github.com/xaviersevillano/EDMA_FDS_analysis_2D.
