# Supplementary material for: Population-specific facial traits and diagnosis accuracy of genetic and rare diseases in an admixed Colombian population
Source: Sci Rep. 2023 Apr 27;13:6869. doi: 10.1038/s41598-023-33374-x (PMC10140286; doi:10.1038/s41598-023-33374-x)
Supplement: Supplementary file 1 — Supplementary Information. [file 41598_2023_33374_MOESM1_ESM.docx]

**Population-specific facial traits and diagnosis accuracy of genetic and rare diseases in an admixed Colombian population**

Luis Miguel Echeverry^1†^, Estephania Candelo^2,3†^, Eidith Gómez^2^, Paula Solís^2^, Diana Ramírez^2^, Diana Ortiz^2^, Alejandro González^4^, Xavier Sevillano^4^, Juan Carlos Cuéllar^5^, Harry Pachajoa^2,3^, Neus Martínez-Abadías^1*^

^1^ Departament de Biologia Evolutiva, Ecologia i Ciències Ambientals (BEECA), Facultat de Biologia, Universitat de Barcelona (UB). Av. Diagonal, 643. Planta 2. 08028 Barcelona (Spain).

^2^ Centro de Investigaciones en Anomalías Congénitas y Enfermedades Raras (CIACER), Universidad ICESI, Cali, Colombia.

^3^ Servicio de Genética, Fundación Clínica Valle del Lili, Cali, Colombia.

^4^ HER - Human-Environment Research Group, La Salle - Universitat Ramon Llull, Barcelona, Spain.

^5^ Universidad ICESI, Cali, Colombia.

^†^ These authors contributed equally

* Corresponding author: [neusmartinez@ub.edu](mailto:neusmartinez@ub.edu)

**SUPPLEMENTARY INFORMATION**

**Automatic facial landmarking**

To perform an automatic registration of 2D landmarks on the frontal image of each individual in the sample, we compared the outputs of three open-source software libraries that offer automatic facial landmark detection on 2D images: Dlib (King, 2009), CLandmarks (Uricar et al., 2016) and LandmarksPy.

A qualitative comparison between these algorithms was applied to facial images taken at different distances, lighting conditions, and head pose orientations. Results revealed significant differences in their accuracy. Dlib library provided an accurate detection of facial landmarks (Fig. S1a); whereas Clandmarks (Fig. S1b) and LandmarksPy (Fig. S1c) suffered from severe landmarking errors, as they did not correctly detect the position of anatomical points around the eyes, lips, or cheeks under different face position and scale conditions (Fig. S1). Therefore, the Dlib landmark detector was the implementation of choice in this study.


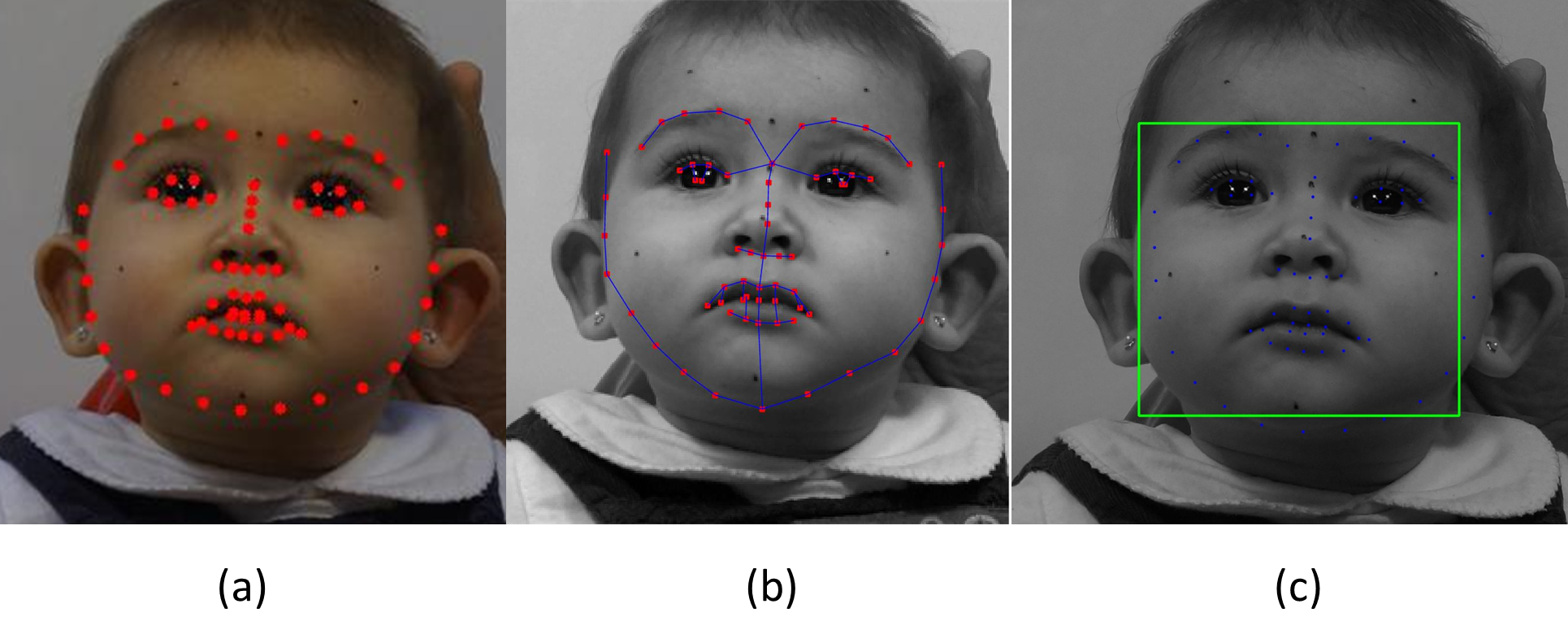


**Figure S1**. Results of automatic detection of 2D facial landmarks using (a) Dlib, (b) Clandmarks, and (c) LandmarksPy.

The landmarking model included in Dlib involves a cascade of regressors trained using the gradient boosting tree algorithm (Kazemi and Sullivan, 2014), and a large database of normative facial images. Dlib is retrainable and constitutes the state-of-the-art for automatic facial landmark detection on 2D images (Bannister et al., 2020). By default, Dlib detects 68 facial landmarks (Figure S1a). We developed a method to automatically obtain the set of 18 facial landmarks used in our study without need of re-training Dlib (Table S1).

**Supplementary Table 1.** Anatomical definition of facial landmarks used in morphometric and statistical analyses to quantify dysmorphologies associated to genetic and rare disorders.

| **Landmark** | **Anatomical Position** |
| --- | --- |
| 1 | Glabella: midpoint between the eyebrows on the median plane |
| 2 | Sellion: deepest point of the nasal root |
| 3 | Pronasale: most anterior point of the nose tip |
| 4 | Subnasale: point where the nasal septum meets the philtrum |
| 5 | Labiale Superius: midpoint of the vermilion seam of the upper lip |
| 6 | Labiale Inferius: midpoint of the vermilion seam of the lower lip |
| 7 | Endocathion R: point in the internal lateral commissure of the right eye |
| 8 | Endocathion L: point in the internal lateral commissure of the left eye |
| 9 | Palpebrale inferius R: most inferior medial point of the lower right eyelid |
| 10 | Exocanthion R: point in the external lateral commissure of the right eye |
| 11 | Palpebrale inferius L: most inferior medial point of the lower left eyelid |
| 12 | Exocanthion L: point in the external lateral commissure of the left eye |
| 13 | Subalare R: the facial insertion of the right alar base |
| 14 | Subalare L: the facial insertion of the left alar base |
| 15 | Chelion R: point located in the right labial commissure |
| 16 | Crista philtra R: crossing of the vermilion line and elevated margin of the right philtrum |
| 17 | Crista philtra L: crossing of the vermilion line and elevated margin of the left philtrum |
| 18 | Chelion L: point located in the left labial commissure |

From the whole set of landmarks provided by Dlib, 15 landmarks directly matched our configuration. The coordinates of the three remaining landmarks were estimated by direct computations between the landmarks coordinates automatically returned by Dlib: the glabella was computed as the central point between the innermost points located in the eyebrows, whereas the palpebrale inferius landmarks of the right and left eyes were computed as the midpoint between the two central lower eyelid landmarks.

**Accuracy of automatic vs manual landmarking**

To verify the validity of the anatomical points automatically detected by the automatic method, the resulting coordinates were compared with the anatomical points registered manually by an expert facial morphologist. To perform this comparison, we used a subset of 40 facial images, including 20 control and 20 syndromic subjects (5 of each syndrome represented in our sample: Down (DS), Morquio (MS), Noonan (NS) and Neurofibromatosis type 1 (NF1)).

We computed the average root mean square error (RMSE) between the coordinates of the manually annotated and the automatically registered landmarks on the control and the syndromic samples. We analyzed the results separately to evaluate the reliability of the automatic landmarking algorithm for registering landmarks on syndromic facial phenotypes, since Dlib is trained on large samples of control individuals.

The average RMSE was 1.75 mm on the control sample, and 1.96 mm on the syndromic sample. Considering that in biological anthropology studies a measurement error below 2 mm is accepted for craniometric measurements (Stull et al., 2014), the precision of the automatic detection method of anatomical points was thus validated on both samples.

Figure S2 presents the average RMSE between the manual and automatic landmarking results for each landmark. In the control sample, five out of 18 landmarks presented a RMSE higher than 2 mm, which corresponded to landmark 1 (glabella), 13 and 14 (left and right subalares), 15 and 18 (left and right chelions). In the syndromic sample, seven landmarks presented a RMSE > 2 mm: landmarks 1 to 4 (glabella, nasion, pronasale and subnasale), 6 (labiale inferius), 11 (left palpebrale inferius) and 15 (left chelion). However, all errors were always below 3.5 mm and affected type-II landmarks (Fig. S2), which are inherently associated with larger measurement errors. These landmarks are defined considering the geometry of the anatomical structure (i.e. as points of local maxima or minima curvature), and are more prone to error, especially in frontal 2D images in which the depth dimension is lost.

**Figure S2**. Average root mean square error (RMSE, measured in millimeters between the manual and automatic landmarking analyzed for each individual landmark for the control and the syndromic subjects.

For a qualitative evaluation of the accuracy of automatic landmarking, Figure S3 presents the manually annotated and the automatically detected facial landmarks for a control individual, and for individuals diagnosed with each of the disorders represented in the syndromic sample. No large deviations were detected. Finally, after automatic landmarking, the coordinates used in the study were visually inspected to discard gross errors before performing the EDMA and FDS analyses.


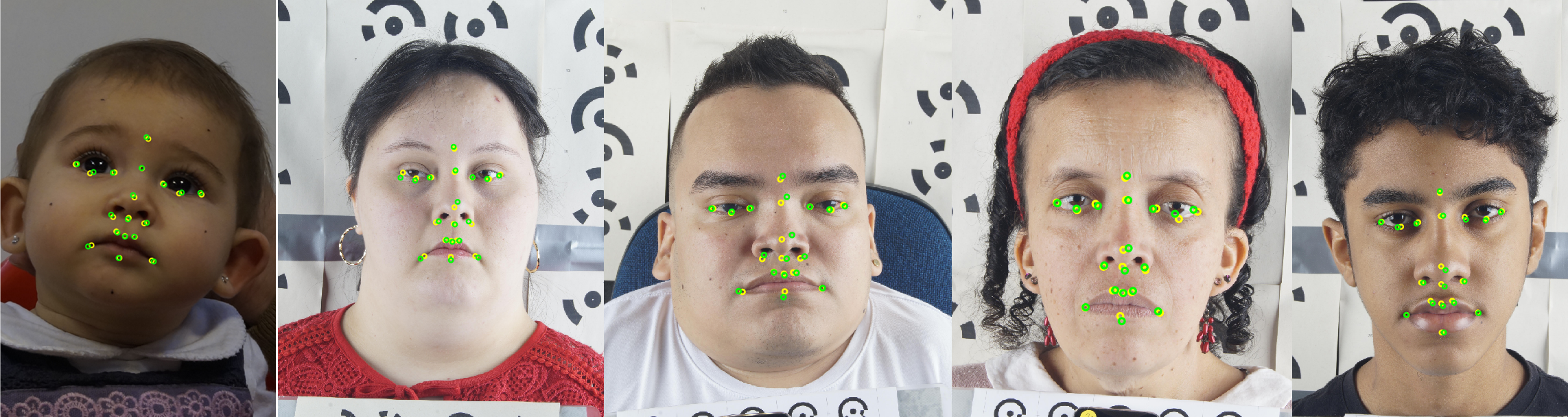


**Figure S3**. Manually annotated landmarks (yellow circles) and automatically detected landmarks (green circles) on control and syndromic individuals of the sample.

**Genetic variants in Morquio syndrome**

In the Colombian sample, we detected four genetic variants (p.Gly301Cys, p.Arg386Cys, p.Arg94Cys, p.Gly333Asp, and p.Ser80Leu) that are missense mutations commonly found in the Colombian population (Pachajoa et al., 2021) (Table S2).

**Supplementary Table 2.** Detailed sample composition of Morquio syndrome patients, including sex (Male (M) / Female (F)), age and genetic mutation associated with Morquio diagnosis.

| **Patient ID** | **Sex** | **Age** | **Genetic Variant** | |  |
| --- | --- | --- | --- | --- | --- |
|  |  |  |  |  |  |
|  |  |  | **Allele 1** | **Allele 2** |  |
| 1 | M | 25 | p.Arg386Cys | p.Gly301Cys |  |
| 2 | F | 24 | p.Ser80Leu | p.Gly301Cys |  |
| 3 | M | 11 | p.Gly301Cys | p.Gly301Cys |  |
| 4 | F | 8 | NA | NA |  |
| 5 | F | 18 | p.Arg386Cys | p.Arg386Cys |  |
| 6 | M | 26 | p.Arg386Cys | p.Arg386Cys |  |
| 7 | F | 7 | p.Gly301Cys |  |  |
| 8 | F | 8 | p.Gly301Cys | p.Gly301Cys |  |
| 9 | M | 13 | p.Arg94Cys | p.Gly333Asp |  |
| 10 | M | 17 | p.Arg94Cys | p.Gly333Asp |  |
| 11 | M | 12 | NA | NA |  |

**REFERENCES**

Bannister, J. J., Crites, S. R., Aponte, J. D., Katz, D. C., Wilms, M., Klein, O. D., Bernier, F. P. J., Spritz, R. A., Hallgrímsson, B., & Forkert, N. D. (2020). Fully Automatic Landmarking of Syndromic 3D Facial Surface Scans Using 2D Images. *Sensors*, *20*(11), 3171. <https://doi.org/10.3390/s20113171>

Kazemi, V., & Sullivan, J. (2014). One millisecond face alignment with an ensemble of regression trees. *Proceedings of the 2014 IEEE Conference on Computer Vision and Pattern Recognition*, pp. 1867-1874. <https://doi.org/10.1109/CVPR.2014.241>

King, D.E. (2009). Dlib-ml: A machine learning toolkit. *Journal of Machine Learning Research*, 10:1755-1758.

Pachajoa, H., Acosta, M. A., Alméciga-Díaz, C. J., Ariza, Y., Diaz-Ordoñez, L., Caicedo-Herrera, G., Cuartas, D., Nastasi-Catanese, J. A., Ramírez-Montaño, D., Silva, Y. K., Moreno, L., Satizabal, J., García, N., Montoya, J., Prada, C., Porras, G., Velasco, H., & Candelo, E. (2021). Molecular characterization of mucopolysaccharidosis type IVA patients in the Andean region of Colombia. *Am. J. Med. Gen.* *Part C*. **187**, 388–395. <https://doi.org/10.1002/ajmg.c.31936>.

Stull, K.E., Tise, M.L., Ali, Z., & Fowler, D.R. (2014) Accuracy and reliability of measurements obtained from computed tomography 3D volume rendered images. *Forensic Science International*, 238:133–140. <https://doi.org/10.1016/j.forsciint.2014.03.005>

Uricar, M., Franc, V., Thomas, D., Sugimoto, A., & Hlavac, V. (2016). Multi-view facial landmark detector learned by the Structured Output SVM. *Image and Vision Computing*, 47:45-59. <https://doi.org/10.1016/j.imavis.2016.02.004>
